# Measuring the oxygen content of a single oil droplet†
†Electronic supplementary information (ESI) available. See DOI: 10.1039/c6sc02357f



**DOI:** 10.1039/c6sc02357f

**Published:** 2016-06-27

**Authors:** Ann Feng, Wei Cheng, Richard G. Compton

**Affiliations:** a Department of Chemistry , Physical & Theoretical Chemistry Laboratory , Oxford University , South Parks Road , Oxford , OX1 3QZ , UK . Email: richard.compton@chem.ox.ac.uk ; Fax: +44 (0)1865275410

## Abstract

Using toluene droplets as a model for artificial oxygen carriers, the real-time measurement of attomole oxygen contents at the individual droplet level is reported for the first time. Oxygen reduction is observed within single individual toluene droplets when they impact with an electrode held at a suitable potential. The oxygen content within single toluene droplets is quantified by referencing the reductive charge from oxygen reduction to the charge from oxidation of a ferrocene filled toluene droplet of known concentration.

## Abstract 2

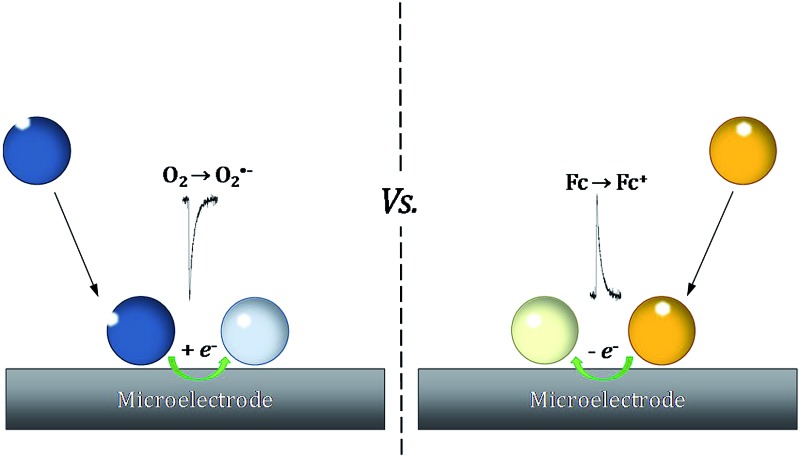
Using toluene droplets as a model for artificial oxygen carriers, the real-time measurement of attomole oxygen contents at the individual droplet level is reported for the first time.

## 1. Introduction

Dissolved oxygen concentration is an important factor in both metabolism and environmental applications including aquaculture. Oxygen is essential for aerobic organisms and the amount of oxygen is a key indicator in living systems, where for example, the oxygen concentration in tumour cells is lower than in normal cells because of poor oxygen supply due to an impaired vascular network.^1^ Current measurement technologies are concerned with bulk samples.^2,3^ However the real-time determination of oxygen content in living systems including within oxygen containing biological carriers such as cells or vesicles at single carrier level is potentially very important and could have wide applications. Optical or electrochemical sensing have been used for oxygen determination in oxygen carriers such as living cells, however the necessary labelling of optical dyes or penetrating nano-sized electrodes may compromise the viability. Non-invasive measurement is challenging.^4–6^ Fluorinated species forming emulsions to “carry” oxygen recently have attracted significant interest as artificial blood^7,8^ but the determination of the oxygen content has not been reported at single droplet levels.

Nano-impact chronoamperometry is a novel method recently developed to characterise single particles, where by virtue of Brownian motion individual nanoparticles randomly collide with an electrode held at a suitable potential to induce quantitative oxidation or reduction of the nanoparticle, or mediated reaction *via* electron transfer through the nanoparticle.^9–11^ Starting with the work of Hellberg *et al.* where lecithin liposomes were recorded through capacitative ‘spikes’,^12^ recently, this method was extended by our group and others to detect “soft” nanoparticles including micelles,^13^ liposomes,^14^ droplets,^15–18^ vesicles,^19,20^ and, it is claimed, enzymes.^21^

In this report, using a toluene emulsion system, we show oxygen reduction within single individual toluene droplets when they impact with an electrode. We then quantify the oxygen content within single toluene droplets by referencing the reductive charge from oxygen reduction to the charge from oxidation of a ferrocene filled toluene droplet of known concentration. Using the toluene droplet as model system for an artificial oxygen carrier, we herein report the first real-time measurement of oxygen contents at the individual droplet level. Moreover, in addition to synthetic blood the oxygen reduction reaction (ORR) is a process at the heart of energy transformation technologies. The enhanced ORR rate at a fuel-cell cathode using emulsions to promote oxygen solubility is recognised.^22–24^

## 2. Results and discussion

**Fig. 1:**
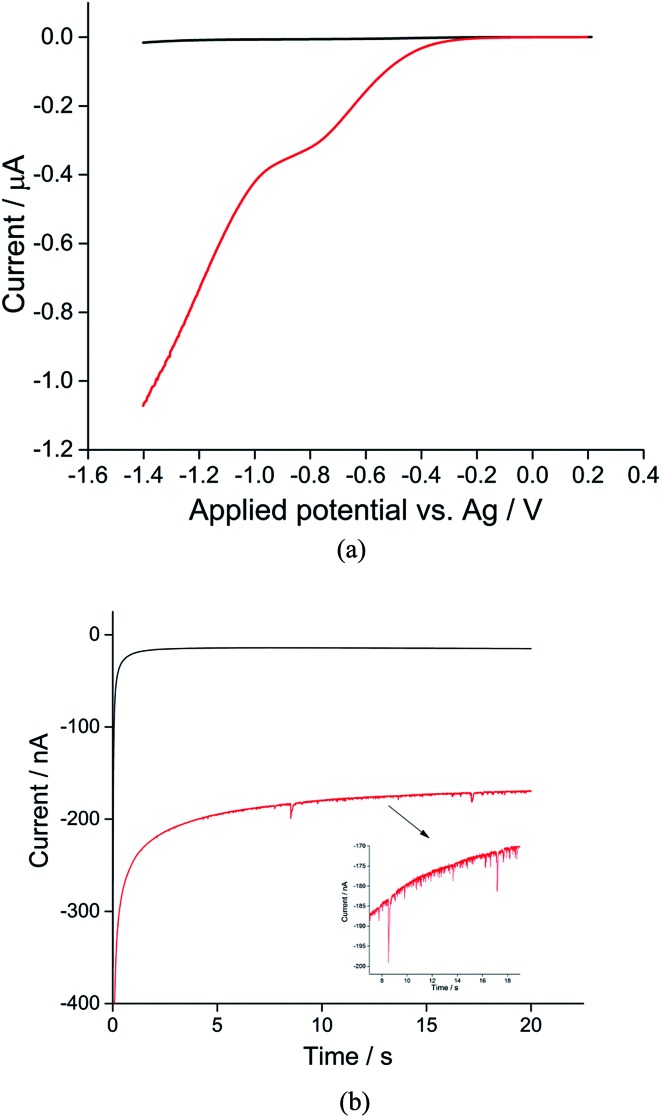
(a) Linear sweep voltammograms and (b) chronoamperograms at –1.4 V *vs.* silver pseudo-reference electrode of the toluene emulsion taken in the nitrogen-degassed (black) and oxygenated (red) states using a microwire electrode. If cyclic voltammetry is recorded in (a) the reverse scan is essentially superimposed on the forward scan.

The synthesis of toluene/water emulsion used is detailed in the Experimental section. It is composed of toluene droplets containing 400 mM trihexyltetradecylphosphonium bis(trifluoromethylsulfonyl)imide ionic liquid. To oxygenate the droplets, the toluene droplet emulsion was bubbled with pure oxygen for at least 10 minutes. Linear sweep voltammograms were then recorded in the oxygenated toluene emulsion using a microcarbon wire electrode.^25^ Comparison of voltammograms collected in the presence or absence of O_2_ showed a clear well-defined reduction peak near –0.7 V *versus* the silver wire pseudo-reference electrode showing the net oxygen reduction from both the ensemble of toluene droplets and from dissolved oxygen (Fig. 1a, red line). A control experiment was carried out in an oxygen-free emulsion. Linear sweep voltammograms were again collected but no oxygen reductive peak was observed (Fig. 1a, black line), indicating that the observed reductive peak corresponds to reduction of oxygen from the toluene emulsion droplets. Other control experiments were carried out in the oxygenated solvents in the absence of droplets; no oxygen reductive peak was observed (Fig. S1†).

Previous studies on oxygen reduction in aprotic^26^ and ionic liquid solvents^27,28^ suggested that molecular oxygen is reduced to superoxide: (O_2_ + e^–^ → O_2_˙^–^), with possible further reduction of the superoxide anion to the peroxide anion (O_2_ + e^–^ → O_2_˙^–^ + e^–^ → O_2_^2–^), in most ionic liquid electrolytes.^29^ In pure phosphonium-based ionic liquids one electron reduction of oxygen is widely claimed,^29^ although the possibility of the superoxide anions becoming protonated in the solvent system with the formation of HO_2_^–^ has been noted.^30^ In the latter case a two electron process forming HO_2_^–^ would become possible. In the toluene/phosphonium-based ionic liquids mixture it seems likely a one electron process dominates reflecting the high proportion of toluene in the droplet solvent but the possibility of a two electron process cannot be rigorously concluded.

For nano-impact experiments, a clean microcarbon wire (length 1 mm, diameter 7 μm) electrode^25^ was held at negative potentials. Under potentiostatted conditions (between –0.6 V and –1.4 V), reductive spikes from individual droplets were observed. A typical chronoamperometric profile of reductive faradaic spikes of individual droplets at –1.4 V *vs.* a silver wire pseudo-reference electrode is shown in Fig. 1b. A control experiment was conducted at a potential of –1.4 V with no droplets in the solution; no spikes were detected, suggesting that the occurrence of reductive spikes is due to the random collisions of droplets with the electrode superimposed on the background current seen in Fig. 1a. The onset of spikes were found to be dependent on reductive potentials with no reductive spikes at reductive potentials of –0.6 V or less negative, consistent with that the spikes corresponding to the faradaic reduction of oxygen in the droplets.

Analogous nanoimpacts experiments were conducted for the oxygen-free system where oxygen was eliminated and no spikes are observed by the nanoimpacts method (Fig. 1b, black), consistent with the notion that the spikes observed under oxygen saturated emulsions correspond to faradaic reduction of oxygen within droplets. It was also found that, at potentials of –1.4 V with a 1 mm long microwire electrode (diameter 7 μm), a high background (see Fig. 1) and overlapping spikes made the integration of individual spikes difficult. Hence for the quantitative analysis of oxygen reduction, chronoamperometric data was collected on a microdisc electrode rather than a microwire (see Experimental section), for which the electrode area was significantly reduced to lower the size of faradaic background signal from dissolved oxygen.

**Fig. 2:**
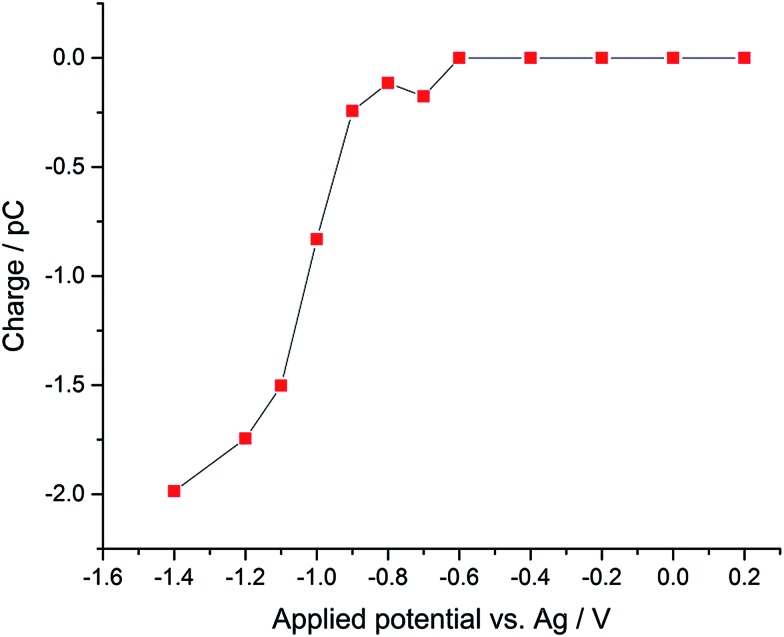
“Voltammogram” of single emulsion droplet constructed from the mean nano-impact charge obtained at a series of potentials using a micro-disk type electrode.

Using potentiostatted conditions between –0.6 V and –1.4 V, reductive spikes from individual droplets were observed (Fig. S2†). The charge resulting from the reduction of individual oxygen containing droplet was then calculated by integrating the area of each spike. A “voltammogram” of single droplet electrochemistry was measured showing the average charge transfer to the droplets as a function of potential, as illustrated in Fig. 2. A “transfer coefficient” *α* ^31,32^ of 0.26 ± 0.03 was derived for oxygen reduction of single emulsion droplet by finding the slope of Tafel plot for the “voltammogram” of the single droplets, while the voltammogram of the bulk emulsions gives an estimated transfer coefficient *α* of 0.21 ± 0.03 (Fig. S3†). Value of *α* significantly below 0.5 are typical for the one electron reduction of O_2_.^28,33,34^ A clear potential shift of *ca.* 0.5 V is seen for complete reduction of oxygen filled droplets as compared to the voltammogram seen for the ensemble of droplets. The increased overpotential is partly due to potential shifts arising from the use of a pseudo-reference electrode, and partly to the greatly increased mass transport seen for a single droplet, which shows convergent diffusion as compared to the more approximately linear diffusion seen for large number of particles within an ensemble.^35^

**Fig. 3:**
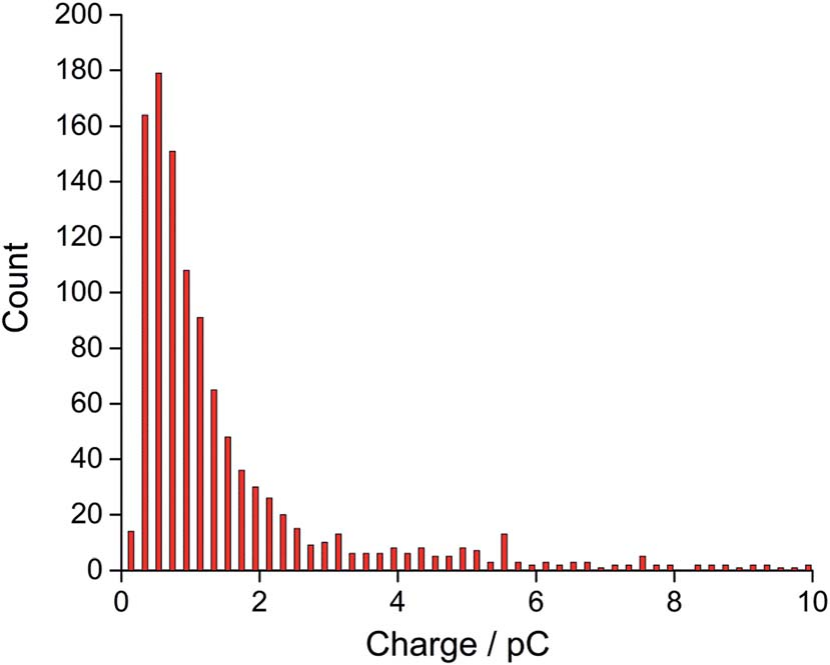
Distribution of charge of the nano-impact spikes observed in toluene emulsion droplets at –1.4 V *vs.* silver pseudo-reference electrode.

The average charge was observed to reach a plateau at a negative potential, indicating the complete reduction of oxygen to superoxide within a single droplet at more negative potentials such as –1.4 V when they impact the electrode. A total of 1130 spikes were recorded at –1.4 V, corresponding to reduction of oxygen filled 1130 single droplets (Fig. S4†). The distribution of charge resulting from oxygen reduction in single droplets gives a modal charge (*Q*_oxygen_) of 0.4 pC (Fig. 3), equivalent to oxygen content of 4.1 attomole per single droplet, assuming one-electron oxygen reduction to form superoxide.^26,29^

**Fig. 4:**
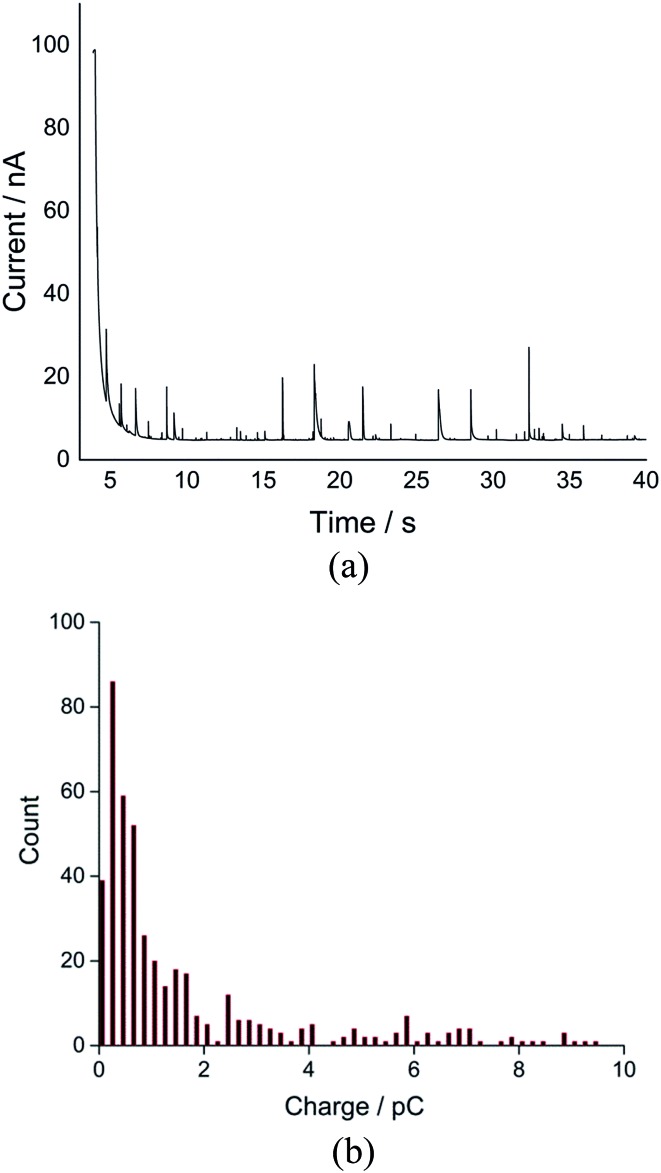
(a) A representative chronoamperometric profile and (b) the charge distribution corresponding to the oxidation of individual ferrocene-containing toluene droplets at +0.7 V *vs.* silver pseudo-reference electrode.

To quantitatively measure the oxygen concentration of single toluene droplets, a known concentration of ferrocene (20 mM) was dissolved in the droplet instead of oxygen by using the same synthesis conditions as before but with the addition of ferrocene (Experimental section). Ferrocene is soluble in toluene but insoluble in water. Hence, a ferrocene droplet reference system was developed where ferrocene is only present in the oil drop and not in the aqueous phase. Nanoimpacts experiments were then performed for single ferrocene droplets. Under potentiostatted conditions, clear oxidative (faradaic) current spikes at 0.7 V *versus* silver were observed (Fig. 4a). This potential is held significantly more positive than the oxidation potential of ferrocene,^36^ ensuring the complete oxidation of the ferrocene inside the droplet.

The charge resulting from oxidation of individual ferrocene containing droplet was calculated by integrating the area of each spike (Fig. 4b). The modal size of the droplets *D*_d_ was derived from the modal charge of 0.3 pC to be *ca.* 700 nm using eqn (1).1
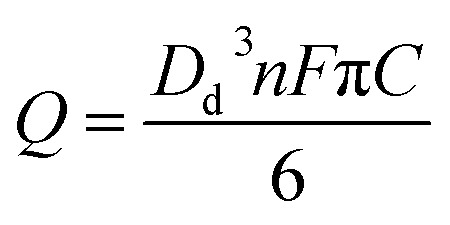
where *Q* is measured charge, *F* is the Faraday constant, and *C* is the concentration of ferrocene in the droplet, the parameter *n* is the number of electrons transferred per molecule during oxidation (*n* = 1 for ferrocene^37^), *N* is the Avogadro constant, *M* is the molar mass of ferrocene.

This inferred size is in good agreement with the size measured through independent dynamic light scattering (DLS) analysis (Fig. S5†), validating the complete oxidation of ferrocene droplet when they impact the electrode, similar to independent observations from a previous study on ferrocene containing droplets.^18^

Next, the oxidative modal charge (*Q*_ferrocene_ = 0.3 pC, Fig. 4b) from the oxidation of ferrocene (*C*_ferrocene_) filled toluene droplets of known concentration when they impact the electrode was then compared to the reductive modal charge obtained from oxygen reduction (*Q*_oxygen_) to estimate the oxygen concentration in single toluene droplets to be 27 mM (eqn (2)), assuming that a one-electron reduction form superoxide from oxygen as discussed above, and the droplet structure is the same for O_2_ and Fc which is reasonable given the dilute concentrations of O_2_ and Fc within the droplets.2
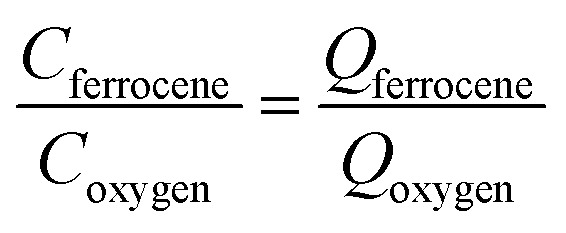



The estimated oxygen concentration in the emulsion droplets is a little higher than the value in bulk toluene reported previously (about 10 mM),^38^ which is likely due to the solubility being promoted by the presence of the ionic liquid. Note that the above analysis assumes the one-electron reduction of oxygen to superoxide. If a two electron process occurs then the concentrations of oxygen inside the droplet would be *ca.* 13.5 mM.

**Fig. 5:**
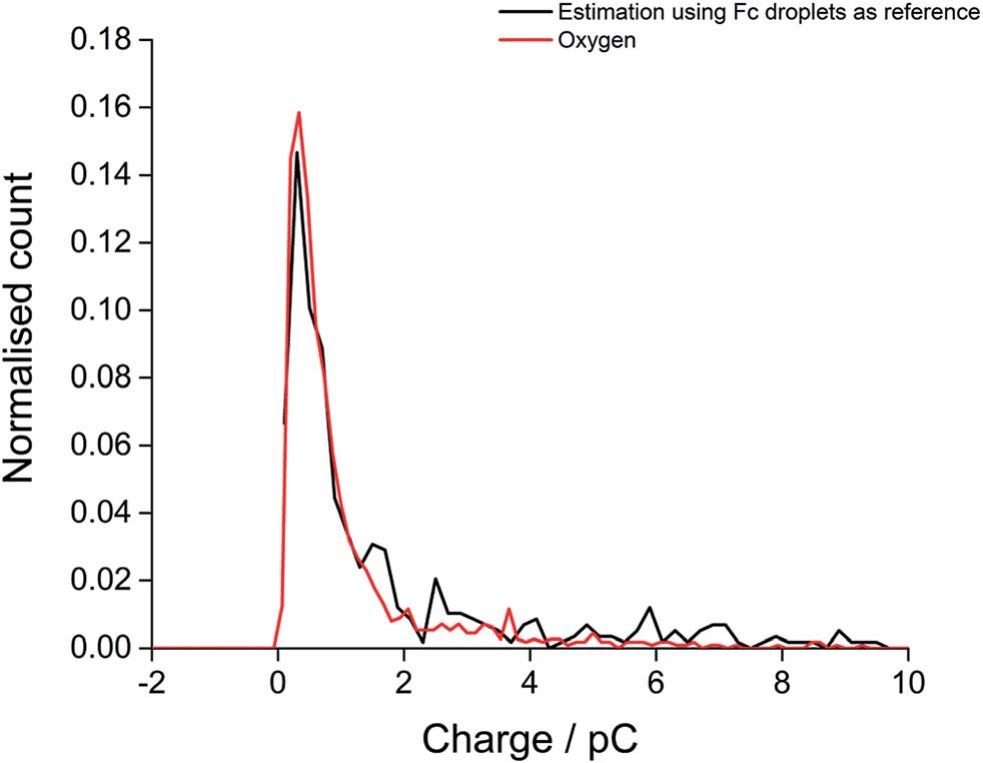
The distribution of experimental charge obtained from oxygen reduction of individual toluene droplets (red) and the charge distribution from theoretical estimation (black) using eqn (2) (*C*_oxygen_ = 27 mM).

Finally, the oxygen content value within single droplets was further validated taking into account the size distribution of the droplets. The distribution of charge obtained from oxidation of the 20 mM ferrocene reference droplets was normalised assuming a concentration of 27 mM, corresponding to the measured oxygen concentration (eqn (2)). The near-identical charge distributions for the O_2_ and Fc droplets after normalisation (Fig. 5) further suggests that quantitative analysis of oxygen content and concentration at the single, individual droplet level is feasible using ferrocene droplets as a reference system.

To conclude, in this report we have shown the use of nano-impacts for measuring and quantifying the oxygen content in an emulsion at the single droplet level. To our knowledge this is the first time that real-time measurement and quantification of oxygen within a single oil droplet has been demonstrated. We believe that this strategy will have major applications in the quantification of oxygen or oxygen related species in cells, vesicles, or artificial oxygen carriers at single carrier level. This simple procedure uniquely facilitates real-time characterization of oxygen carrier at single carrier level, which is impossible by other methods.

## 3. Experimental section

### Materials

Toluene (analytical reagent grade, Fischer Scientific) and ferrocene (Fc, 98%, Sigma-Aldrich) were each used as received; trihexyltetradecylphosphonium bis(trifluoromethylsulfonyl)imide ([P6,6,6,14][NTf2], >98%) was acquired from Iolitech, Germany and used as received. The water used was ultrapure water (Millipore, resistivity = 18.2 MΩ cm) and nitrogen and oxygen were obtained from oxygen-free, BOC Gases plc. Finally carbon microfibers (diameter 7 μm) were obtained from (Goodfellow Cambridge Ltd.).

### Synthesis and characterisation of toluene microdroplets

The toluene-in-water emulsion was synthesized as described elsewhere.^18^ Briefly, [P6,6,6,14][NTf2] was dissolved in toluene to a concentration of 400 mM. Then 100 μl of the mixture was added dropwise to 5 ml of vigorously-stirred water, and allowed to stir for 20 s. Sonication was applied using 6 cycles of 7 s on, 3 s off pulses. For the oxygen oxygenated droplets, the toluene droplet emulsion was oxygenated by bubbling with oxygen for 10 minutes. For the oxygen-free emulsion, the same synthesis was carried out using nitrogen-degassed toluene and water, using a sealed container for both the stirring and sonication phases. The synthesised emulsion was degassed using nitrogen for a further 10 minutes before experiments.

Ferrocene-containing toluene droplets were synthesised by an analogous method: 20 mM of ferrocene was dissolved in toluene along with 400 mM ionic liquid in the first stage. The mixture was then stirred and sonicated analogously. The size distribution of Fc-containing toluene droplets were characterised by Dynamic Light Scattering (DLS, Malvern Instruments Ltd, UK).

### Electrochemistry of the toluene droplets

Experiments were carried out in a typical three-electrode setup in a Faraday cage. The potentiostat used was an Autolab PGSTAT30 (Metrohm-Autolab BV, Utrecht, The Netherlands). The working electrodes were either homemade carbon microwires of 7 μm diameter and approximately 1 mm length, or a similar homemade carbon microdisc electrode of 7 μm diameter and approximately 0 mm length.^25^ A silver wire was used as a quasi-reference electrode while the counter electrode was a platinum foil. All experiments were thermostatted to 25 ± 1 °C.

Nanoimpact spikes were counted and integrated using the program “Signal Counter” (developed by Dr D. Omanović, Center for Marine and Environmental Research Zagreb, Croatia).^39^ Spikes were also checked manually to prevent the counting of signals due to electrical noise, and to check that integrations were taken from the centre of the signal baseline.
