# Supplementary material for: Measuring the oxygen content of a single oil droplet
Source: Chem Sci. 2016 Jun 27;7(10):6458–62. doi: 10.1039/c6sc02357f (PMC5355955; doi:10.1039/c6sc02357f)
Supplement: Supplementary file 1 [file SC-007-C6SC02357F-s001.pdf]

Electronic Supplementary Material (ESI) for Chemical Science.
This journal is © The Royal Society of Chemistry 2016

Supporting Information

Linear sweep voltammograms in oxygenated toluene, oxygenated water, and oxygenated toluene/water emulsion

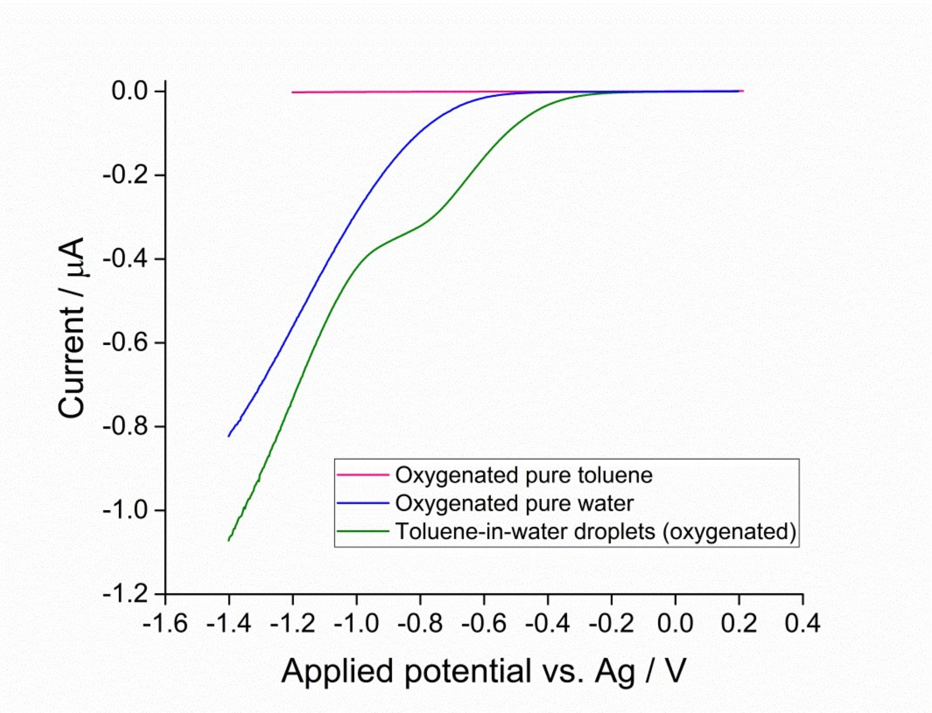

Figure S1 Linear sweep voltammograms in oxygenated pure toluene (red), oxygenated pure water (blue), and oxygenated toluene emulsion using a microwire electrode.

Nanoimpacts of oxygenated toluene emulsion droplets

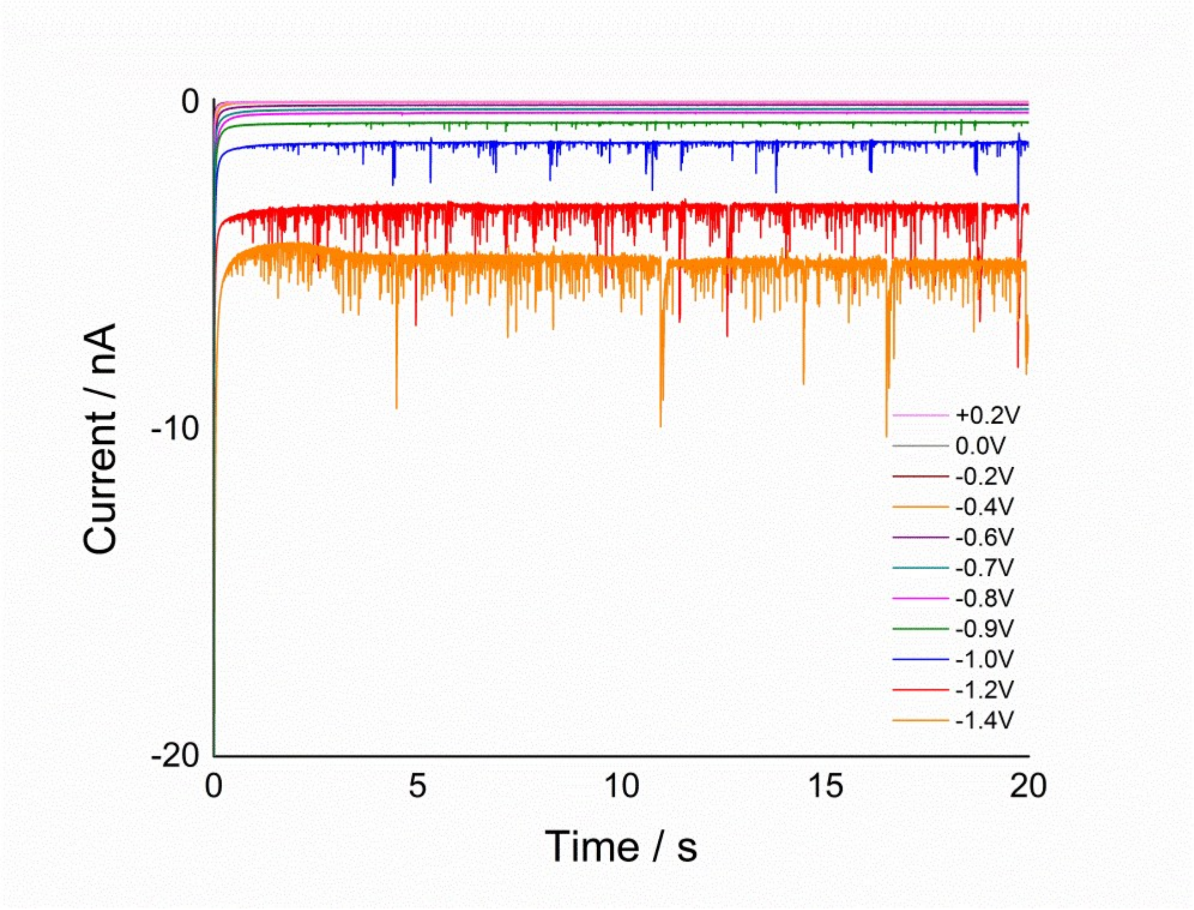

Figure S2 Chronoamperograms collected at a series of negative potentials covering the range of 0.2 V to -1.4 V vs. silver pseudo-reference electrode in the oxygenated toluene emulsion using a microdisk electrode.

Tafel plot of the voltammogram of bulk emulsions and single droplet "voltammogram"

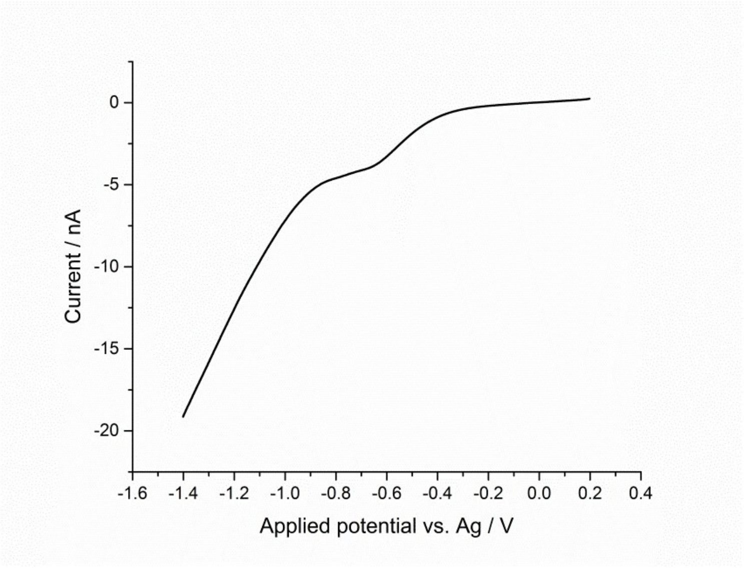

Figure S3 (a) A voltammogram showing the oxygen reduction behaviour in the bulk emulsion

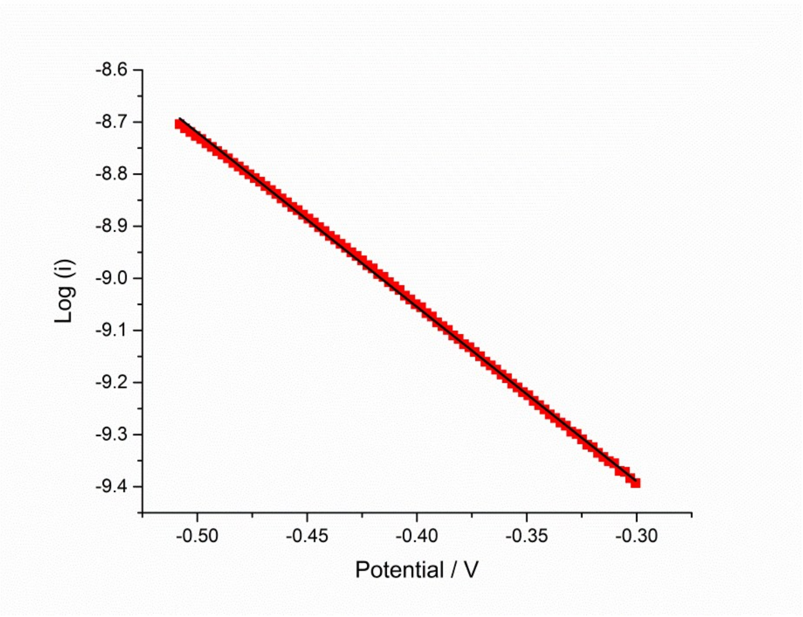

Figure S3 (b) a Tafel plot for oxygen reduction in the bulk emulsion

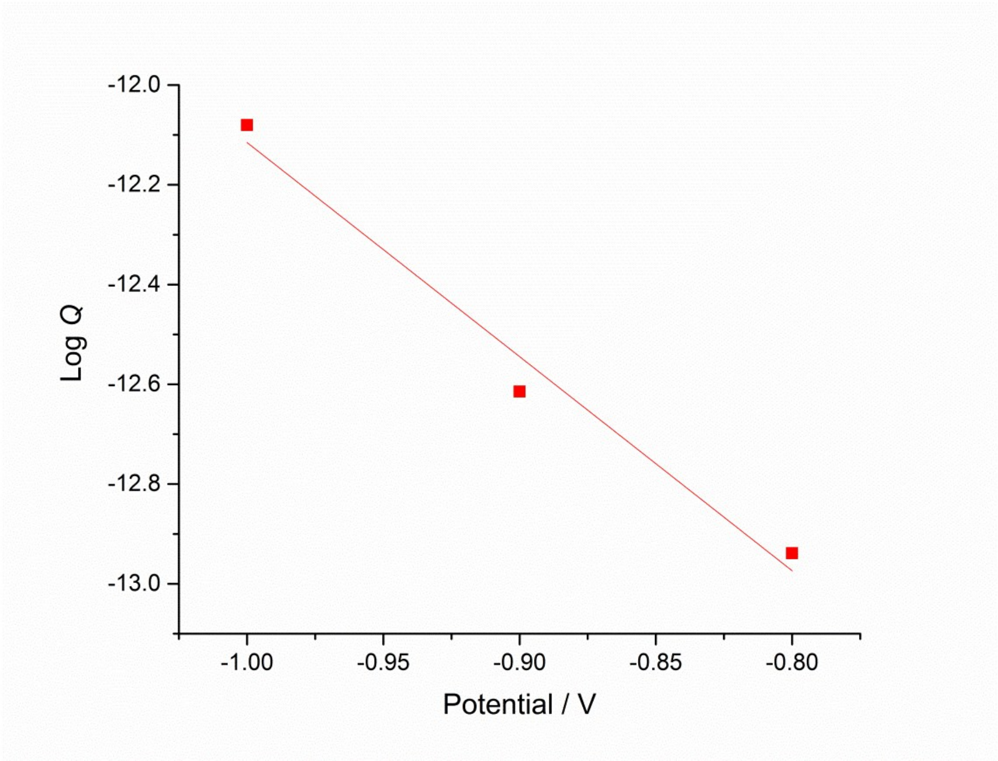

Figure S3 (c) Tafel plot for single droplet “voltammogram”.

Nano- Impacts: the charge resulting from oxygen filled in each single emulsion droplets of a total number of 1130

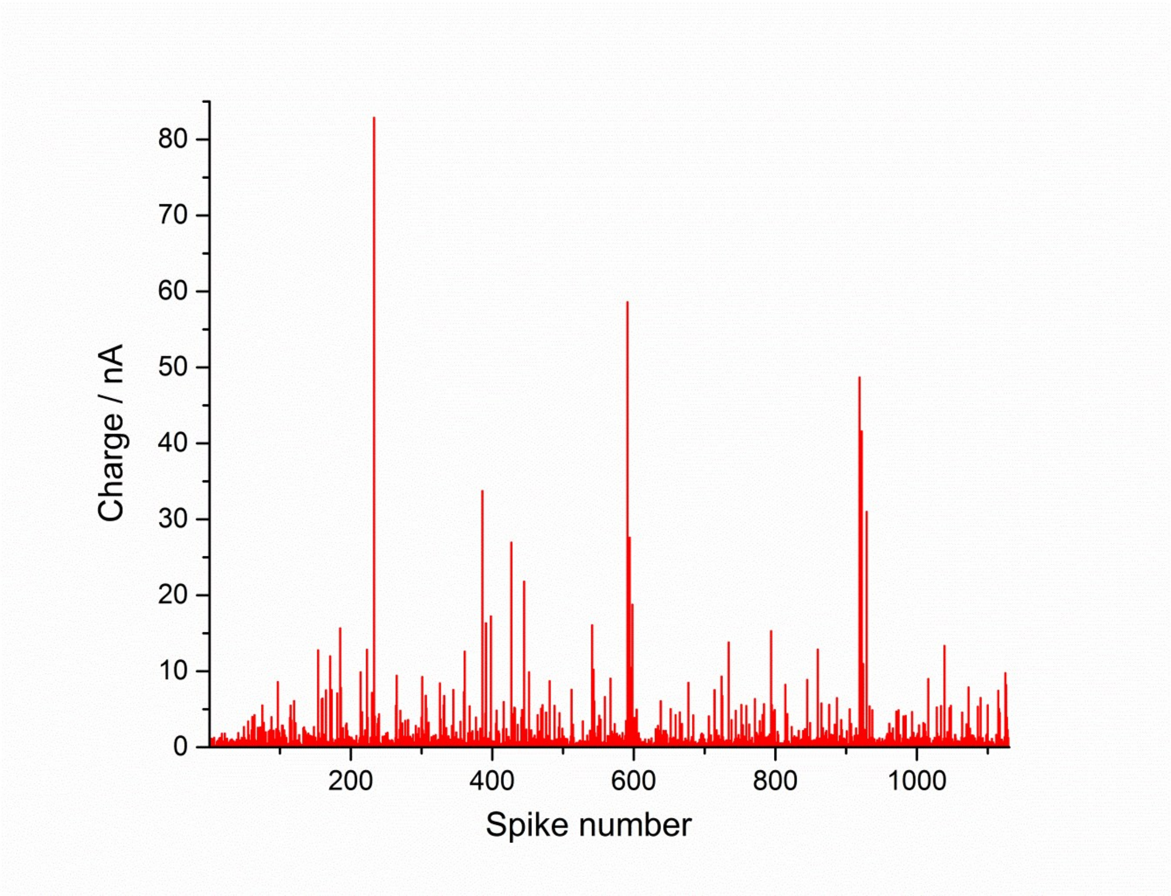

Figure S4 The charge resulting from the reduction of oxygen filled 1130 single droplets (spike number is in the order of appearance during the time course of experiment.

Size distribution of synthesised Fc filled droplets

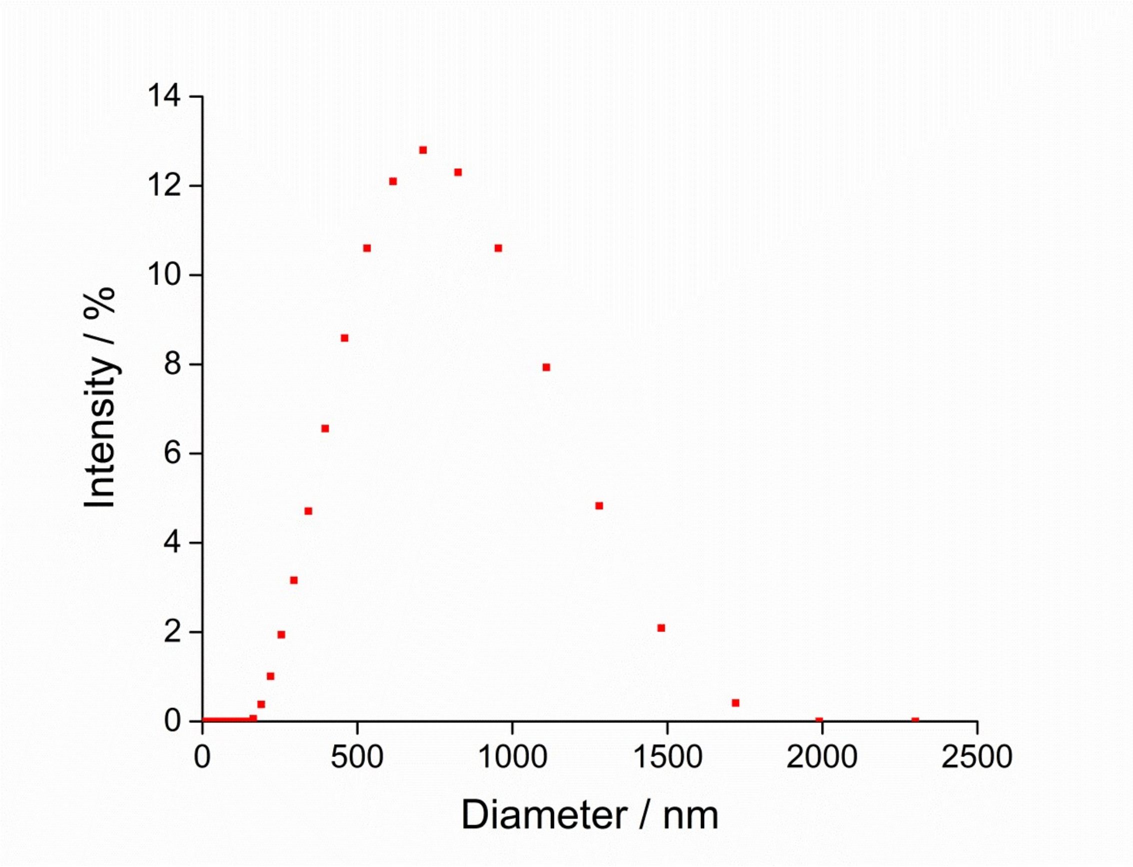

Figure S5 The size distribution of synthesised Fc filled droplets measured by DLS.
